# Supplementary material for: Repeated Social Defeat Enhances CaCl2-Induced Abdominal Aortic Aneurysm Expansion by Inhibiting the Early Fibrotic Response via the MAPK-MKP-1 Pathway
Source: Cells. 2022 Feb 19;11(4):732. doi: 10.3390/cells11040732 (PMC8870675; doi:10.3390/cells11040732)
Supplement: Supplementary file 1 [file cells-11-00732-s001.zip › cells-1539756-supplementary.pdf]

Repeated social defeat enhances $CaCl_2$-induced abdominal aortic aneurysm expansion by inhibiting the early fibrotic response via the MAPK-MKP-1 pathway

Supplementary Figure Legends

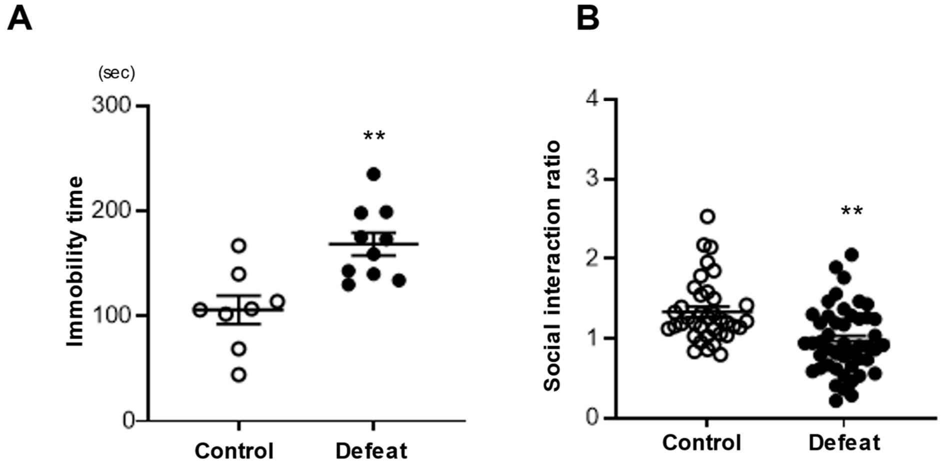

Figure S1. Analysis of depression-like behaviors.

(A) Tail suspension test. Values represent the mean ± standard error of the mean (SEM) for eight non-exposed mice and ten RSD-exposed mice before $CaCl_2$ application. ***p* < 0.01 vs. non-exposed mice; Student's t-test. **(B)** Social interaction test. Values represent mean ± standard error of mean (SEM) for 37 non-exposed mice and 47 RSD-exposed mice before $CaCl_2$ application. ***p* < 0.01 vs. non-exposed mice; Student's t-test. RSD, repeated social stress.

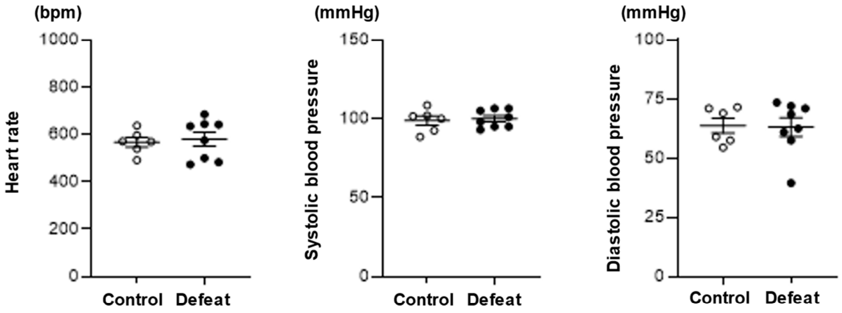

Figure S2. Body weight and hemodynamic parameters before $CaCl_2$ application are comparable between the two groups. Body weight, blood pressure, and heart rate are equivalent between the two groups. Values are mean ± SEM for six control and eight defeated mice. Control: undefeated control mice. Defeat: defeated mice.

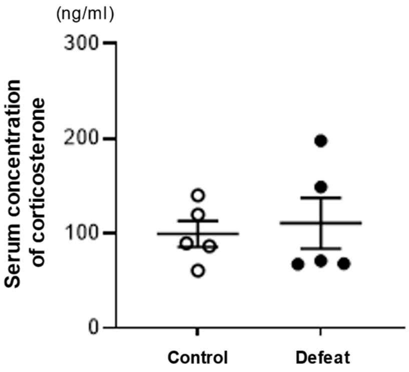

Figure S3. Serum concentrations of corticosterone. Values are mean ± SEM for five control and five defeated mice. Control: undefeated control mice. Defeat: defeated mice.

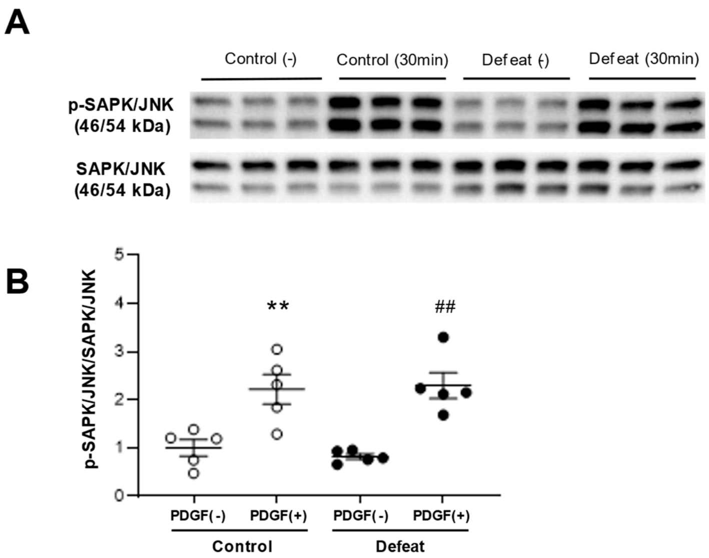

Figure S4. Repeated social defeat does not affect PDGF-induced JNK phosphorylation in primary cultured VSMCs. (A and B) Representative Western blot of JNK phosphorylation in VSMCs at 30 min after PDGF stimulation and a quantitative analysis of the protein expression. Values are the mean ± SEM for five control and five defeated mice. **$p < 0.01$ vs. control without PDGF stimulation. $^{\#\#}p < 0.01$ vs. defeat without PDGF stimulation; two-way repeated measures ANOVA with the Tukey–Kramer post hoc test.

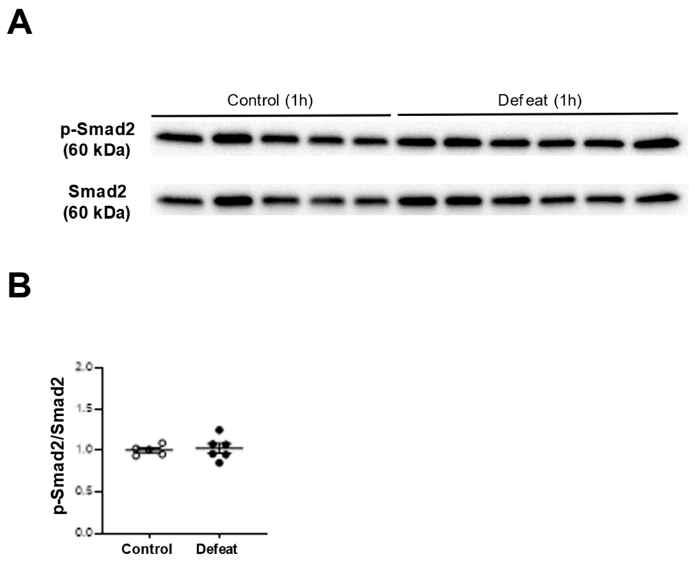

Figure S5. Repeated social defeat does not affect TGF-β-induced Smad2 phosphorylation in primary cultured VSMCs. (A and B) Representative Western blot of Smad2 phosphorylation in VSMCs at 60 min after TGF-β stimulation and a quantitative analysis of the protein expression. Values are the mean ± SEM for five control and five defeated mice.

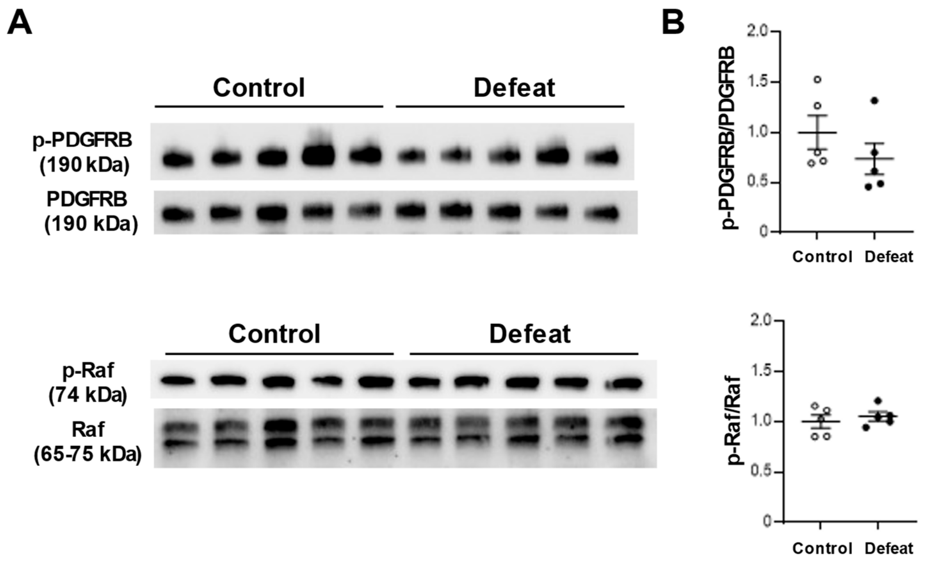

Figure S6. Repeated social defeat does not affect PDGF-induced phosphorylation of PDGFRB and Raf in primary cultured VSMCs. (A and B) Representative Western blot of the phosphorylation of platelet derived growth factor receptor β (PDGFRB) and Raf in VSMCs at 30 min after PDGF stimulation and a quantitative analysis of the protein expression. Values are the mean ± SEM for five control and five defeated mice.

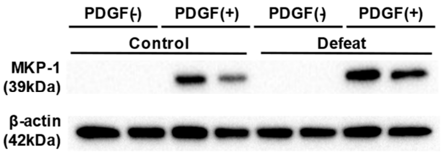

Figure S7. MKP-1 protein expression before PDGF stimulation. Representative Western blot of MKP-1 in VSMCs before and after PDGF stimulation.
